# Integrated Flavoromics and Metabolomics Reveal Aroma and Bioactive Characteristics of Six Mainstream Citri Reticulatae Pericarpium Cultivars

**DOI:** 10.3390/foods15122090

**Published:** 2026-06-10

**Authors:** Luxin Xie, Yanjing Dong, Qian Qin, Cheng Jiang, Lifeng Shang, Kailin Qiao, Bo Wang, Jinxiang Zeng, Quanpeng Luo, Lingyun Zhong, Shouwen Zhang

**Affiliations:** 1 Research Center for Traditional Chinese Medicine Resources and Ethnic Minority Medicine, Jiangxi University of Chinese Medicine, Nanchang 330004, China; xlx3021@163.com (L.X.); gfq1106@163.com (Q.Q.); zjx@jxutcm.edu.cn (J.Z.); 2 Pharmacy School, Jiangxi University of Chinese Medicine, Nanchang 330004, Chinaly1638163@163.com (L.Z.); 3 Jiangxi Key Laboratory for Sustainable Utilization of Chinese Materia Medica Resources, Jiangxi University of Chinese Medicine, Nanchang 330004, China

**Keywords:** Citri Reticulatae Pericarpium, aroma characterization, volatile organic compounds, terpenoids, flavonoids, antioxidant

## Abstract

Citri Reticulatae Pericarpium (CRP), a traditional functional food with various cultivars, is widely used in spices and food additives. However, systematic comparisons of flavor and chemical profiles across mainstream cultivars remain lacking. This study systematically analyzed the flavor characteristics, volatile/non-volatile metabolic profiles, and antioxidant activity of six mainstream CRP cultivars. A total of 455 volatile organic compounds (VOCs) were identified, among which terpenoids showed distinct cultivar-specific accumulation and strongly affected aroma; 35 key VOCs were screened as pivotal contributors to distinct aroma profiles. Untargeted metabolomics identified 1289 non-volatile metabolites, among which polymethoxyflavones and flavonoid glycosides exhibited significant cultivar-specific accumulation. The six CRP cultivars showed five distinct metabolic accumulation patterns, which significantly affected the differences in bioactive compound accumulation and flavor formation among different cultivars. This study reveals the aroma formation basis and metabolic features of mainstream CRP cultivars, offering insights into CRP functional food development and genetic breeding.

## 1. Introduction

Citri Reticulatae Pericarpium (CRP), also known as Chenpi, is derived from the dried ripe peel of citrus fruits. It is a traditional functional food ingredient and has a long history in the cooking and dietary habits of East Asia [1]. CRP is highly valued for its unique aroma and rich flavor, which underpin its applications in teas, seasonings, candies, and functional food formulations, widely used as a flavor enhancer, dietary antioxidant, and source of phytonutrients [2]. Meanwhile, some inherent bioactive components in CRP, including essential oils, polyphenols, and flavonoids, exhibit excellent antioxidant activity and play important roles in addressing various diseases [3,4]. Driven by the development of the global healthcare industry, particularly the prominent performance of CRP in COVID-19 treatment and immune enhancement, the economic output of CRP—especially representative local varieties such as Guangchenpi (CRP from Guangdong)—is growing rapidly with the support of local governments [5,6].

Citrus ranks among the earliest domesticated fruits. Long-term regional agricultural practices and artificial selection have shaped distinctive citrus landraces, resulting in geographically differentiated CRP with varied flavor and chemical profiles [7]. In this study, six representative CRP cultivars originating from major producing regions across China were selected, including *C. reticulata* ‘Chachi’ (CZG), *C. reticulata* ‘Dahongpao’ (DHP), *C. reticulata* ‘Unshiu’ (WZM), *C. reticulata* ‘Tangerina’ (FJ), *C. reticulata* cv. Zhangshuensis (ZTH), and *C. reticulata* cv. Sanhuhongju (HJ) [8]. These cultivars cover core CRP-producing provinces (Guangdong, Sichuan, Zhejiang, Fujian, and Jiangxi) and exhibit distinct morphological, aromatic, and phytochemical traits (summarized in Table S1). CZG and DHP are distinctive cultivars mainly distributed in Guangdong and Sichuan provinces, respectively [9,10]. Extensive studies have addressed their cultivation practices and pharmacological activities, and CZG has long been recognized as the most therapeutically effective CRP cultivar [6,11,12]. WZM is a representative Zhejiang-origin cultivar and also one of the primary ancestral genotypes for many modern citrus varieties [13]. FJ is a Fujian-native cultivar with over 300 years of cultivation history; it is morphologically similar to DHP but remains under-investigated [14]. ZTH is a Jiangxi-indigenous cultivar with a unique flavor profile distinct from other CRP types. HJ is another Jiangxi-endemic cultivar characterized by intense floral aroma and abundant unique flavonoids, suggesting promising pharmacological and nutritional value [8]. Collectively, these geographically diverse cultivars provide a sound basis for revealing regional flavor differentiation and metabolite diversity in CRP.

Regional differences in flavor and phytochemical composition among diverse CRP cultivars remain largely unclear, and systematic profiling of their characteristic metabolites is essential to reveal the chemical basis of these variations. Metabolomics serves as a powerful tool for compound detection and is widely applied in diverse fields such as agriculture, medicine, and materials science. Currently, it has become a core approach for elucidating food components, functional factors, and the effects of processing [15,16,17]. In the development of functional foods, researchers leverage this technology to comprehensively characterize bioactive small molecules in foods and food-medicinal plants, identifying metabolite clusters with potential anti-inflammatory, antioxidant, or gut microbiota-regulating properties [18]. For food authenticity and traceability, metabolomics enables the construction of “metabolic fingerprints” for specific producing areas or cultivars, facilitating the identification of adulterated products and geographical indication products [19]. Additionally, in studies on metabolite changes during postharvest preservation, thermal processing, or fermentation of fruits and vegetables, metabolomics can simultaneously monitor the dynamics of hundreds to thousands of compounds, providing data support for optimizing processing technologies and retaining nutritional components [20,21,22,23]. Owing to its characteristics of high coverage, high accuracy, and high throughput, metabolomics has emerged as a crucial bridge linking basic plant metabolism research and food nutrition applications. Thus, metabolomics can effectively identify metabolic differences between different CRPs, laying a reliable data foundation for the subsequent screening of flavor-characteristic substances and characteristic compounds.

Flavoromics further complements metabolomics by focusing specifically on volatile and semi-volatile aroma compounds, which directly determine sensory quality [17,24]. Combined multi-omics approaches integrating metabolomics, flavoromics, and lipidomics have been widely used to identify flavor biomarkers and their biosynthetic pathways [25,26]. When combined with sensory evaluation and electronic sensing techniques, these methods bridge chemical composition and perceived flavor performance [27,28]. Among available platforms, GC-MS-based, widely targeted metabolomics enables sensitive, high-throughput profiling of volatile metabolites in complex plant matrices, which is particularly suitable for CRP aroma research [29,30].

Despite extensive research focusing on individual CRP cultivars, systematic multi-omics comparisons among geographically representative CRP varieties remain largely unexplored, limiting our understanding of their diverse functional potential. Therefore, this study integrated GC-MS-based widely targeted metabolomics and LC-MS-based untargeted metabolomics to characterize volatile and non-volatile metabolites across six typical CRP cultivars. Multivariate statistical analyses, including principal component analysis (PCA) and orthogonal partial least squares-discriminant analysis (OPLS-DA), were used to identify cultivar-specific metabolic signatures. Meanwhile, sensory evaluation, electronic tongue analysis, and antioxidant activity assays were performed to correlate chemical variation with sensory quality and functional properties. This work aims to elucidate cultivar-dependent flavor-formation mechanisms and antioxidant characteristics, providing theoretical support for the targeted development of high-value CRP functional food products and raw material screening.

## 2. Materials and Methods

### 2.1. Plant Materials

**Figure 1:**
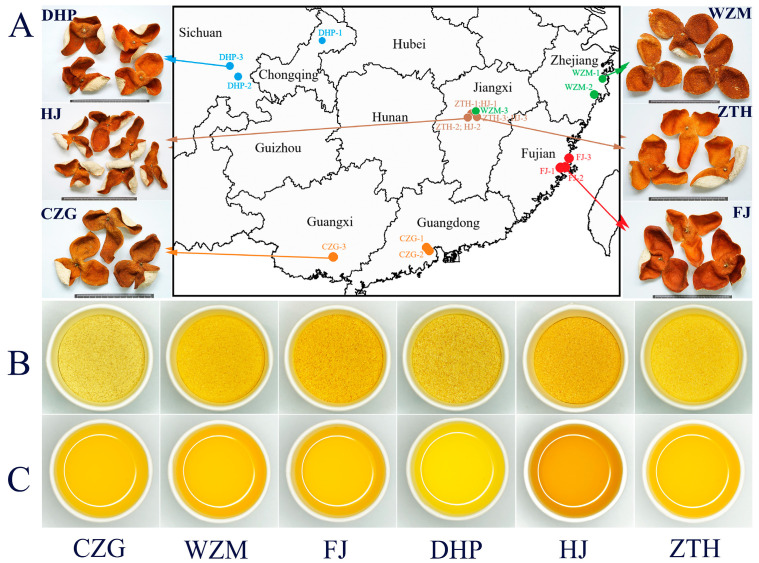
Geographical origin and morphological characteristics of six CRP cultivars. (**A**) The collection sites of different CRP cultivars. (**B**) Powder characteristics of different CRP cultivars. (**C**) Filtrate from the six CRP cultivars after brewing.

Six CRP cultivars (CZG, DHP, FJ, WZM, HJ, ZTH) were collected from major production regions between November 2023 and January 2024. Three independent orchards were sampled for each cultivar to ensure biological replication (Figure 1A, Table S1). Fresh citrus fruits (50–100 kg per orchard) were randomly harvested and thoroughly washed. Peels were manually separated from the pulp and processed by conventional sun drying, followed by indoor storage and repeated sun drying in the following spring and autumn. Finally, 25–30 intact peels from each sample were further dried at 40 °C, ground into powder, and sieved through a 40-mesh sieve. The powder was stored at −80 °C until analysis.

### 2.2. Chemicals and Reagents

Sodium chloride (NaCl), potassium chloride (KCl), and tartaric acid were purchased from Shanghai Macklin Biochemical Technology Co., Ltd. (Shanghai, China). Formic acid was obtained from Aladdin Reagent Co., Ltd. (Shanghai, China). Analytical-grade ethanol was supplied by Xilong Scientific Co., Ltd. (Shantou, China). n-Hexane, methanol, and acetonitrile were purchased from Merck KGaA (Darmstadt, Germany). The GC-MS internal standard was acquired from Sigma-Aldrich (St. Louis, MO, USA).

### 2.3. Sensory Evaluation

Sensory aroma evaluation was conducted by 20 trained panelists (12 males, 8 females, mean age 27.05 years) according to the sensory evaluation for Pubei Chenpi (T/CAI 041-2023 [31]) with minor modifications. Briefly, 5.0 g of dried CRP sample was cut into small pieces, steeped in 250 mL boiling water for 5 min, and the infusion was used for aroma assessment. Each sample was assigned a unique three-digit code and randomly presented to the panelists for evaluation after brewing. Panelists reached a consensus that the characteristic aromas of the samples could be described using five sensory attributes: fresh, herbal, floral, orange-like, and citrus-like. In addition, the “fresh” attribute was further described by sub-features associated with sensory perceptions of phenolic, sulfurous, spicy, spoiled, and gasoline-like substances that may induce neural stimulation. The intensity of each aroma attribute was rated on a 0–10 scale, where 0 indicated no or negligible intensity and 10 represented extremely strong intensity. Each panelist evaluated each sample three times on different days. The final results were expressed as mean scores.

### 2.4. GC-E-Nose Analysis

We referred to the method described by Yuan et al., with minor modifications [32]. Approximately 2 g of sample was weighed and placed into a 20 mL headspace vial, followed by incubation at 35 °C for 10 min. Sample detection was performed using a GC-E-nose (Heracles NEO, Alpha MOS, Toulouse, France). The injection volume was 250 μL, the injector temperature was 200 °C, the flow rate was 125 μL/s, and the injection time was 7 s. All samples were adsorbed on the trap at 40 °C, transferred to two types of chromatographic columns (MXT-5 and MXT-1701) at a flow rate of 10 mL/min, and desorbed at 240 °C with a flow rate of 1.0 mL/min. The column temperature was initially set at 50 °C, then ramped up to 80 °C at a rate of 1.0 °C/s, further increased to 250 °C at a rate of 3.0 °C/s, and held for 21 s. The data acquisition time was 110 s, and the FID gain was 12.

### 2.5. Electronic Tongue Evaluation

The taste characteristics of different CRP cultivars were analyzed using the SA402B electronic tongue system (INSSENT, Atsugi, Japan). The sensor array consists of two AgCl reference electrodes and six taste-specific sensors (CAO, NAO, AE1, AAE, CTO, and GL1), capable of detecting six basic taste profiles: sour, bitter, astringent, umami, salty, and sweet. In addition, the system can quantify secondary taste attributes, including astringency aftertaste (Aftertaste-A), bitterness aftertaste (Aftertaste-B), and richness. The reference solution used in the analysis was composed of 0.3 mmol/L tartaric acid and 30 mmol/L KCl. The positive electrode solution was prepared as 0.1 mol/L KCl + 0.01 mol/L KOH + 30% (*v*/*v*) ethanol, while the negative electrode solution contained 0.1 mol/L HCl + 30% (*v*/*v*) ethanol. The internal filling solution for the electrodes and sensors was 3.33 mol/L KCl + 10 mg/L AgCl. The sample soup solution was prepared as follows: 2.0 g of the sample powder was weighed and mixed with 100 mL of deionized water preheated to 40 °C. The mixture was ultrasonicated for 20 min, then transferred to a 50 mL centrifuge tube and centrifuged at 5000 rpm for 4 min. The supernatant was collected and used as the test solution for analysis. The detection conditions were set as follows: prior to measurement, the taste sensors and ceramic reference electrode were cleaned for 330 s and equilibrated for 30 s. Each sample was measured for 30 s to assess the primary taste profile, followed by a 30 s aftertaste measurement. Each sample was analyzed in triplicate to ensure data reproducibility.

### 2.6. HS-SPME-GC-MS Analysis

A total of 500 mg of sample powder was transferred into a 20 mL headspace vial (Agilent, Palo Alto, CA, USA). Subsequently, 2 mL of saturated sodium chloride (NaCl) solution and 20 μL of internal standard solution (10 μg/mL) were added, and the vial was sealed immediately. For solid-phase microextraction (SPME) analysis, each vial was equilibrated at 60 °C for 5 min under continuous agitation. Volatile organic compounds (VOCs) were then extracted using a 120 μm DVB/CWR/PDMS fiber (Agilent) at 60 °C for 15 min. After extraction, the fiber was inserted into the injection port of a gas chromatograph (GC, Model 8890, Agilent), and the VOCs were thermally desorbed in splitless mode at 250 °C for 5 min. Chromatographic separation and compound identification were performed using an Agilent 8890 GC coupled with an Agilent 7000D mass spectrometer (MS). The separation was achieved on a 30 m × 0.25 mm × 0.25 μm MD-5 ms capillary column (5% phenyl polymethyl siloxane). Helium was used as the carrier gas at a constant linear velocity of 1.2 mL/min, and the injection port temperature was maintained at 250 °C. The oven temperature program was set as follows: initial temperature of 40 °C (held for 3.5 min), ramped to 100 °C at 10 °C/min, then increased to 180 °C at 7 °C/min, and finally elevated to 280 °C at 25 °C/min (held for 5 min). Mass spectrometric analysis was conducted in electron impact (EI) ionization mode at 70 eV. The temperatures of the quadrupole mass analyzer, ion source, and transfer line were set to 150 °C, 230 °C, and 280 °C, respectively. Compounds were identified and quantified using selected ion monitoring (SIM) mode.

### 2.7. Determination of Main Flavonoids by HPLC

For flavonoid quantification, 200 mg of CRP powder was extracted with 25 mL of methanol under ultrasonic treatment for 45 min. After standing, the supernatant was filtered through a 0.22 μm membrane filter. Analysis was performed on a Shimadzu LC-20AT system (Shimadzu, Kyoto, Japan) equipped with an Agilent Eclipse XDB-C18 column (250 mm × 4.6 mm, 5 μm). The mobile phase consisted of 0.1% formic acid in water (A) and acetonitrile (B) with gradient elution: 0–10 min, 20–30% B; 10–25 min, 30–42% B; 25–37 min, 42–70% B; 37–40 min, 70% B. The flow rate was 1.0 mL/min, column temperature 30 °C, and injection volume 10 μL. Hesperidin was detected at 283 nm, and other polymethoxyflavones were detected at 330 nm.

### 2.8. Intargeted Metabolomics Analysis by LC-MS

A total of 50 mg of sample powder was accurately weighed using an electronic balance (MS105DU, Mettler Toledo, Zurich, Switzerland), followed by the addition of 1200 μL pre-cooled 70% methanol aqueous solution (−20 °C) for extraction. The mixture was vortexed for 30 s every 30 min, and this extraction process was repeated six times. After extraction, the sample was centrifuged at 12,000 rpm for 3 min. The supernatant was carefully collected, filtered through a 0.22 μm microporous membrane, and transferred into a sample vial for UPLC-MS/MS analysis.

UPLC analysis was performed on a Shimadzu LC-30A system (Shimadzu, Japan). Chromatographic separation was achieved using a Waters ACQUITY Premier HSS T3 column (1.8 μm, 2.1 mm × 100 mm). The mobile phases consisted of 0.1% formic acid in water (solvent A) and 0.1% formic acid in acetonitrile (solvent B). The gradient elution program was set as follows: 5–20% B (0–2 min), 20–60% B (2–5 min), 60–99% B (5–6 min), isocratic elution at 99% B (6–7.5 min), re-equilibration from 99% to 5% B (7.5–7.6 min), and 5% B (7.6–10 min). The column temperature was maintained at 40 °C, with a flow rate of 0.4 mL/min and an injection volume of 4 μL. Identical gradient conditions were applied for chromatographic separation in both positive and negative ionization modes.

Mass spectrometry analysis was carried out using a Triple TOF 6600+ mass spectrometer (SCIEX, Framingham, MA, USA) in both positive and negative ionization modes. Information-dependent acquisition (IDA) mode was employed, with the electrospray ionization (ESI) source temperature set to 550 °C. The ion source gas (Gas1) and curtain gas (CUR) pressures were maintained at 50 psi and 25 psi, respectively. The declustering potential (DP) was 60 V (positive mode) and −60 V (negative mode), while the ion spray voltage floating (ISVF) was 5000 V (positive mode) and −4000 V (negative mode). The TOF-MS parameters were configured as follows: MS1 scan range of 50–1000 Da with an accumulation time of 200 ms; MS/MS fragment ion scan range of 25–1000 Da with an acquisition time of 40 ms per spectrum. The collision energy (CE) was set to 30 V (positive mode) and −30 V (negative mode).

Raw mass spectrometry data were preprocessed using MS-DIAL v5.5 software. Peaks with peak areas below 10,000 and missing values exceeding 80% across all samples were filtered out. Preliminary compound annotation was performed by matching MS/MS spectra against the in-house spectral library (MSMS-public-ExperimentSpectile-VS19). Subsequently, metabolites with low-confidence annotations were removed by comparing their spectral and retention characteristics with those in the in-house database.

### 2.9. Determination of Antioxidant Properties

The 2,2-diphenyl-1-picrylhydrazyl (DPPH) and 2,2′-azino-bis (3-ethylbenzothiazoline-6-sulfonic acid) (ABTS)free radical scavenging assays are commonly used methods for evaluating antioxidant activity and are widely applied in the assessment of the antioxidant potential of plant-derived compounds. In this study, the DPPH Free Radical Scavenging Assay Kit (Beijing Solarbio Science & Technology Co., Ltd., Beijing, China) was employed according to the manufacturer’s instructions. In brief, 0.05 g of the sample powder was weighed and mixed with 1 mL of the extraction solvent. The mixture was vortexed thoroughly and incubated in a water bath at 40 °C for 30 min. Following incubation, the sample was centrifuged at 10,000 rpm for 10 min, and the supernatant was collected and stored in the dark at 4 °C for subsequent analysis. For the DPPH assay, 10 µL of the sample solution was added to 190 µL of freshly prepared DPPH working solution in a 96-well microplate and allowed to react in the dark for 30 min. The absorbance was then measured at 515 nm using a microplate reader. Each sample was analyzed in triplicate. In this assay, 10 mg/mL ascorbic acid (vitamin C) was used as the positive control, yielding a DPPH radical scavenging activity (RSA) of 93.91%.

The ABTS free radical scavenging assay was performed using an ABTS Antioxidant Assay Kit (Beijing Solarbio Science & Technology Co., Ltd., China), following the manufacturer’s instructions. Briefly, 0.05 g of the sample powder was weighed and mixed with 1 mL of the extraction solvent. The mixture was vortexed thoroughly and incubated in a water bath at 40 °C for 30 min. Following incubation, the sample was centrifuged at 10,000 rpm for 10 min, and the supernatant was collected and stored in the dark at 4 °C for subsequent analysis. For the ABTS assay, 10 µL of the sample solution was added to 190 µL of freshly prepared ABTS working solution in a 96-well microplate and allowed to react in the dark for 6 min. The absorbance was then measured at 405 nm using a microplate reader. Each sample was analyzed in triplicate. In this assay, 10 mol/L vitamin C was used as the positive control, yielding an ABTS RSA of 94.86%.

### 2.10. Statistical Analysis

All experimental data were subjected to one-way analysis of variance (ANOVA) using SPSS 26.0 software (IBM Corporation, Armonk, NY, USA), with statistical significance set at *p* < 0.05 and results expressed as mean ± standard deviation (SD). Differential comparison analysis and data visualization were conducted using Origin 2025 (OriginLab, Northampton, MA, USA) and the Metware Cloud platform (https://www.metware.cn/).

## 3. Results and Discussion

### 3.1. Volatile Profiles of Different CRP Cultivars

#### 3.1.1. Volatile Metabolic Profiles Among Different CRP Cultivars

**Figure 2:**
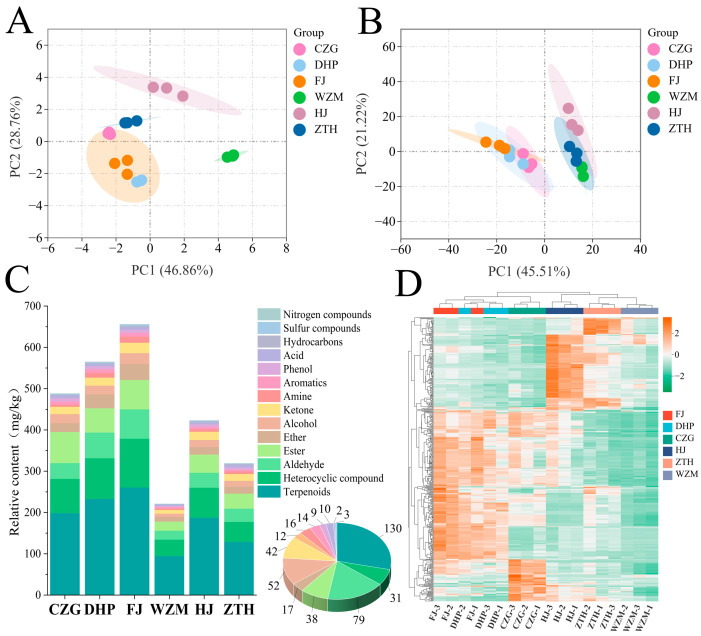
Volatile metabolic profile analysis of six CRP cultivars. (**A**) Principal component analysis of GC-E-nose analysis data. (**B**) Principal component analysis of widely targeted flavor-omics data. (**C**) Distribution of 455 volatile components in six CRP cultivars, the pie chart depicts the statistical number of different types of VOCs. (**D**) Cluster heatmap analysis of the relative content of VOCs in all CRP samples.

Post-aged samples showed obvious differences in morphology, powder color, and infusion color (Figure 1A–C). Infusions of FJ, DHP, and HJ were dark, whereas those of ZTH, CZG, and WZM were lighter and clearer. To distinguish the volatile components of different CRP cultivars, we initially employed a gas chromatography-mass spectrometry electronic nose (GC-MS-E-nose) for rapid detection. Through this approach, 13 compounds were identified across the six cultivars (Table S2), with terpenoid components—including *D*-Limonene, *γ*-Terpinene, Myrcene, and *β*-Pinene—accounting for the largest proportion. Eleven VOCs were detected in CZG, which was consistent with the number identified in ZTH. However, the peak areas of most VOCs in ZTH were lower than those in CZG, with only Myrcene exhibiting relatively higher accumulation levels among the six CRP cultivars. Additionally, we noted that characteristic components (β-Caryophyllene and 2-Tridecanone) were exclusively detected in HJ. By contrast, only 5 VOCs were identified in WZM, most of which were much lower than those in other CRP cultivars. Meanwhile, 7 and 9 VOCs were detected in DHP and FJ, respectively, and the two cultivars exhibited similar VOC accumulation levels. PCA revealed that FJ and DHP shared similar aroma profiles among the six cultivars, while the other four (ZTH, CZG, WZM, and HJ) were scattered in the coordinate space (Figure 2A). These results indicate that the GC-MS-E-nose can serve as a rapid and effective method for distinguishing different CRP cultivars.

However, the metabolites detected by GC-MS-E-nose were relatively limited. Therefore, widely targeted metabolomics and flavoromics were further employed for the comprehensive characterization of VOCs in the six CRP cultivars. Significant differences in volatile metabolic profiles were observed among the different cultivars; however, the results exhibited a certain deviation from those of GC-MS-E-nose. ZTH shared more similar volatile metabolic characteristics with WZM and HJ, while CZG, DHP, and FJ showed comparable component accumulation patterns (Figure 2B). The more comprehensive VOCs dataset generated by this combined approach reveals that the volatile components among different citrus cultivars are relatively close overall. A total of 455 VOCs were identified across the six CRP cultivars. Specifically, 438 were detected in ZTH, 425 in CZG, 441 in DHP, 445 in FJ, 442 in WZM, and 446 in HJ (Table S3). These VOCs were classified into 14 categories, including 130 terpenoids, 79 esters, 52 ketones, 42 alcohols, 38 aldehydes, 31 heterocyclic compounds, 17 ethers, 16 acids, 14 phenols, 12 amines, 10 hydrocarbons, 9 aromatics, 3 nitrogen compounds, and 2 sulfur-containing compounds. Terpenoids, heterocyclic compounds, esters, and aldehydes were identified as the major VOC groups, collectively accounting for more than 75% of the total VOC content (Figure 2C), which is in agreement with previous studies [33]. Although the types and numbers of VOCs were similar among cultivars, notable differences were observed in the total VOC content and relative abundances. Among them, FJ (607.30–712.65 μg/g) and DHP (482.78–595.12 μg/g) exhibited significantly higher VOC levels compared to the other four cultivars. The VOC content in CZG ranged from 391.04 to 583.22 μg/g, in HJ from 328.53 to 490.11 μg/g, and in ZTH from 281.02 to 359.23 μg/g. In contrast, WZM had the lowest total VOC content, ranging from 185.18 to 240.48 μg/g. As shown in the clustering heatmap (Figure 2D), most VOCs were present at higher levels in DHP and FJ, and some characteristic VOCs were uniquely detected in ZTH, HJ, and CZG.

#### 3.1.2. Characteristic Accumulation of Terpenoids in CRP and Its Impact on Characteristic Aroma Formation

**Figure 3:**
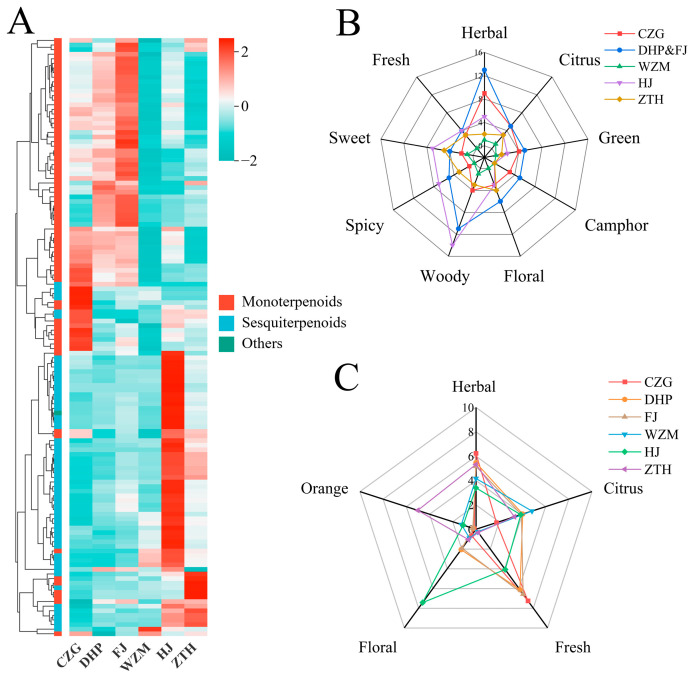
Characteristic terpenoids and flavor characteristics of six CRP varieties. (**A**) Heat map of the relative content of 130 terpenoids in the six CRP varieties. (**B**) Aroma characteristics of characteristic terpenes in six CRP cultivars. (**C**) Aroma characteristics of six CRP cultivars by sensory evaluation.

Terpenoids were the dominant volatile components in CRP, and monoterpenoids accounted for more than 70% of total terpenoids. As the main contributors to aroma, monoterpenoids such as D-limonene showed extremely high levels in most cultivars. Sesquiterpenoids exhibit high structural diversity, accounting for generally 5–30% of total terpenoids [34]. Compared with monoterpenoids, sesquiterpenoids possess greater chemical stability and often outperform monoterpenoids in terms of pharmacological activity intensity, mechanism specificity, and metabolic stability [35]. During the aging process of CRP, monoterpenoids gradually volatilize or oxidize, while the proportion of sesquiterpenoids increases; concurrently, new oxidized sesquiterpenoids are generated, enhancing pharmacological activity [36]. Interestingly, hierarchical clustering analysis revealed distinct specificity in the accumulation of 130 terpenoids among different CRP cultivars (Table S4). Specifically, CZG, DHP, and FJ showed higher accumulation of most monoterpenoids but lower levels of most sesquiterpenoids. In contrast, HJ and ZTH exhibited significantly higher accumulation of most sesquiterpenoids with relatively lower monoterpenoid contents (Figure 3A). Notably, both cultivars originate from the same geographical origin, and HJ is speculated to be one of the parental varieties of ZTH. This genetic relationship may contribute to their similar advantages in the accumulation of sesquiterpenoids.

Further statistical analysis of the aroma descriptions of these characteristic terpenoids indicated that the characteristic terpenoids in CZG collectively contribute to an herbal-citrus-green-woody aroma profile (Figure 3B). Among them, monoterpenoids such as α-terpinolene, o-cymene, β-pinene, and γ-terpinene are characteristically accumulated in CZG and serve as key contributors to its aroma [37]. A total of 41monoterpenoids were relatively more abundant in DHP and FJ, with an overall aroma profile of herbal-woody-floral- spicy-camphor. 13 of these monoterpenoids are described as having herbal notes, 11 contribute to woody aromas, and an additional 6 exhibit floral characteristics. The aroma contribution of sesquiterpenoids in DHP and FJ was less prominent. In HJ, 43 highly accumulated sesquiterpenoids collectively impart a woody-spicy-sweet-floral aroma. α-Caryophyllene, α-cedrene, and germacrene B form the dominant woody aroma; α-zingiberene, cbenene, and isocaryophyllene contribute to the spicy notes; meanwhile, components including geranyl isobutyrate, (+)-valencene, β-elemene, and β-guaiene enhance citrus and sweet aromas.

In ZTH, 5 monoterpenoids and 7 sesquiterpenoids were identified as characteristic terpenoids, collectively exhibiting a multi-layered aroma of sweet-floral-citrus-woody. Floral notes were predominant: citronellol acetate, neryl acetate, β-bisabolol, and β-damascenone contribute floral-sweet-citrus aromas, while copaene and β-caryophyllene impart woody notes. Three characteristic terpenoids were identified in WZM, namely γ-cadinene, and neral, which exhibit herbal and sweet aromas. To verify the consistency between the aroma profiles derived from characteristic terpenoids and the actual sensory perceptions, five key aroma attributes were selected for evaluation (Figure 3C). The aroma characteristics of CZG, DHP, FJ, and HJ were found to be more intense and distinct compared to those of the other cultivars. CZG exhibited a strong, pungent earthy scent with prominent herbal notes, which was consistent with the herbal-woody aroma profile of its characteristic terpenoids. DHP shared a similar aroma profile with FJ, characterized by fresh (metallic-like) and herbal notes, aligning with their dominant herbal-spicy-camphor aromas. HJ was distinguished by a rich, sweet floral aroma, accompanied by fresh and woody undertones, matching the woody-spicy-citrus-floral aroma characteristics of its highly accumulated terpenoids. ZTH presented a unique blend of orange and citrus notes, which was in line with the floral-sweet-citrus-woody aroma derived from its characteristic terpenoids. WZM exhibited a relatively faint aroma, with subtle woody and citrus peel notes, consistent with the weak herbal and sweet aromas of its characteristic terpenoids.

#### 3.1.3. Key VOCs Contributing to Aroma Formation in Different CRP Cultivars

**Figure 4:**
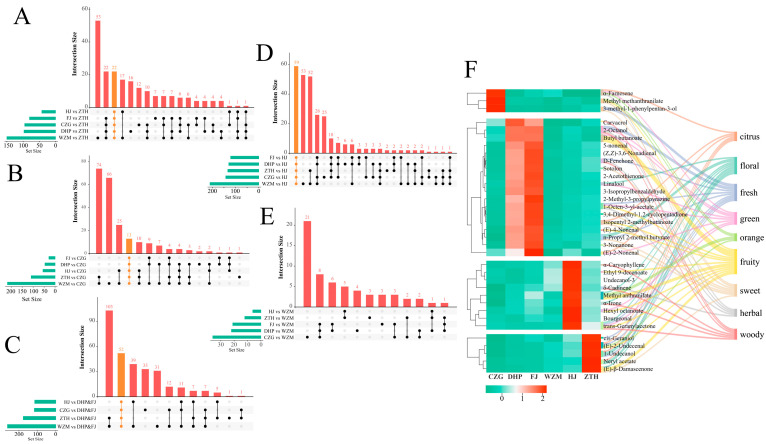
Core aroma compounds of different CRP cultivars. (**A**) Venn analysis for screening commonly upregulated characteristic VOCs in ZTH compared with other CRP. (**B**) Venn analysis for screening commonly upregulated characteristic VOCs in CZG. (**C**) Venn analysis for screening commonly upregulated characteristic VOCs in DHP&FJ. (**D**) Venn analysis for screening commonly upregulated characteristic VOCs in HJ. (**E**) Venn analysis for screening commonly upregulated characteristic VOCs in WZM. (**F**) Heatmap analysis of rOAV and aroma sankey diagram for 35 key VOCs influencing aroma formation. In panels (**A**–**E**), orange bars represent VOCs shared across all comparison groups, while red bars indicate VOCs detected in only a subset of the comparison groups.

To further clarify the differences in volatile components between the six CRP cultivars and identify the key VOCs contributing to the formation of characteristic aromas in CRP cultivars, an OPLS-DA model was constructed for comparative analysis (Figure S1). Differential volatile components were screened using the criteria of Fold Change (FC) ≥ 2 or ≤0.5 and Variable Importance in Projection (VIP) ≥ 1. Between ZTH and CZG, 204 differential VOCs were identified, of which 95 were upregulated in ZTH; between ZTH and DHP, 228 differential VOCs were detected with 97 upregulated in ZTH; between ZTH and FJ, 262 differential VOCs were found, including 80 upregulated in ZTH; between ZTH and WZM, 162 differential VOCs were identified, with 150 upregulated in ZTH; and between ZTH and HJ, 175 differential VOCs were detected, of which 42 were upregulated in ZTH. Venn diagram analysis was performed on these upregulated differential VOCs, revealing 22 commonly occurring compounds—including 5 terpenoids, 4 ketones, and 3 acids. The aroma contribution of individual VOCs was evaluated by the ratio of their concentration to their respective odor thresholds (Figure 4A). VOCs with a relative odor activity value (rOAV) > 1 are generally regarded as key contributors to the characteristic aroma profile of samples [38]. Statistical analysis of the rOAV values of these 22 characteristic VOCs showed that 1-Undecanol, (E)-2-Undecenal, cis-Geraniol, Neryl acetate, and (E)-β-Damascenone had rOAV > 1 (Table S5). These compounds exhibit citrus, fruity, and floral aroma, and are thus identified as the major sources of ZTH’s unique orange-like aroma (Figure 4F).

Furthermore, this study also analyzed the key aroma components contributing to the characteristic aroma of other CRP cultivars. Using the same method, an OPLS-DA model was constructed with CZG as the control group for differential comparison analysis, followed by screening of differential VOCs (Figure S2). Subsequent Venn diagram analysis identified 13 commonly upregulated differential VOCs in CZG, which exhibit fresh, herbal, and citrus aroma notes (Figure 4B). Among these, Methyl methanthranilate, α-Farnesene, and 3-methyl-1-phenylpentan-3-ol showed rOAV > 1, playing crucial roles in shaping the characteristic herbal aroma of CZG (Figure 4F). Recent studies have also reported that Methyl methanthranilate, α-Farnesene, and other related components are key contributors to the aroma formation of CZG [37].

DHP and FJ exhibited similar sensory aroma evaluations. Consistent with this, the present study revealed that they had minimal metabolic differences and similar metabolic profiles. Notably, these two cultivars have been classified as the same variety in previous studies—further supporting their grouping together (DHP&FJ) for comparison with other CRP cultivars. Although FJ contained higher levels of most VOCs than DHP due to geographical differences, this taxonomic consistency (as reported in prior literature) justified their combined analysis [8]. Using DHP&FJ as the control group for differential screening, a total of 52 commonly upregulated VOCs were identified, which exhibit green, fruity, fresh, and sweet aroma notes (Figure 4C and Figure S3). Meanwhile, 18 VOCs were found to have rOAV > 1, namely 3-Isopropylbenzaldehyde, 2-Octanol, Isopentyl 2-methylbutanoate, Butyl butanoate, Carvacrol, 2-Methyl-3-propylpyrazine, 3-Nonanone, D-Fenchone, 1-Octen-3-yl acetate, Sotolon, 3,4-Dimethyl-1,2-cyclopentadione, (E)-4-Nonenal, 2-Acetothienone, Linalool, n-Propyl 2-methylbutyrate, (Z,Z)-3,6-nonadienal, (e)-2-nonenal, and 5-Nonenal. Notably, three aldehydes—(Z,Z)-3,6-Nonadienal, (E)-2-Nonenal, and 5-Nonenal—showed extremely high response values with rOAV > 1000. These compounds primarily impart fatty, green, and cucumber-like aroma notes, which align with the metallic-tinged fresh aroma characteristic of DHP&FJ. Additionally, certain terpenoids (Linalool, Carvacrol, D-Fenchone, and n-Propyl 2-methylbutyrate) exhibit floral and camphor-like aroma notes. Furthermore, esters (Isopentyl 2-methylbutanoate, Butyl butanoate, 1-Octen-3-yl acetate, and Sotolon) contribute citrus and herbal aroma (Figure 4F). Collectively, these compounds synergistically shape the characteristic aroma of DHP&FJ.

In the differential screening with HJ set as the control, a total of 59 commonly upregulated VOCs were identified in HJ. Notably, 32 of these were sesquiterpenoid VOCs—further confirming its distinct advantage in sesquiterpenoid accumulation (Figure S4). Among the 46 components whose odor thresholds have not been evaluated, several high-abundance sesquiterpenoids (e.g., β-Cubebene, δ-Elemene, and Germacrene B) exhibit sweet, fruity, and woody aroma notes (Figure 4D). Only 9 VOCs, namely Ethyl 9-decenoate, α-Caryophyllene, Hexyl octanoate, Bourgeonal, Undecanol-3, Methyl anthranilate, α-Irone, δ-Cadinene, and trans-Geranylacetone, were found to have a rOAV > 1. These compounds exhibit fruity, woody, and floral aroma notes and serve as major contributors to HJ’s sweet floral aroma (Figure 4F).

For subsequent differential screening assays where WZM served as the control, no commonly upregulated VOCs were identified in WZM (Figure S5). Furthermore, WZM did not exhibit prominent accumulation of volatile components (Figure 4E); instead, its volatile metabolic profile was more similar to that of ZTH—an observation indirectly supported by its faint aroma. As a widely distributed cultivar, most modern citrus cultivars for fruit production have been selected and bred from its progeny. Currently, WZM may have been influenced by breeding practices, potentially leading to the degradation of certain characteristic aroma components due to environmental or artificial selection [39].

### 3.2. Non-Volatile Metabolomic Analysis and Its Correlation with CRP Taste Properties

#### 3.2.1. Taste Evaluation Among Different CRP Cultivars

**Figure 5:**
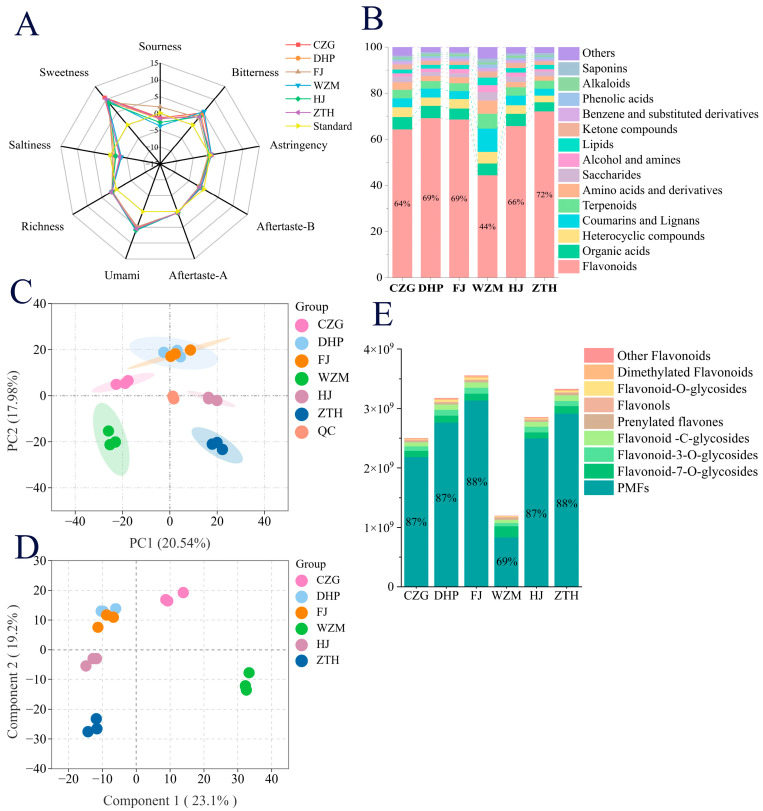
Taste evaluation and non-volatile metabolic profile analysis of different CRP. (**A**) Electronic tongue evaluation. (**B**) The quantities of different types of metabolites and their proportions in CRP. (**C**) The PCA score plot. (**D**) The PLS-DA score plot. (**E**) Classification and peak area statistics of all flavonoids.

To characterize the taste differences among different CRP cultivars, the present study conducted a quantitative evaluation of their taste profiles via electronic tongue analysis (Figure 5A). Among the measured taste attributes, sweetness yielded the highest response values (ranging from 8 to 10), with CZG and ZTH recording the highest scores, whereas FJ and HJ exhibited relatively lower values. In terms of the source of sweetness, the primary driver of this taste attribute in CRP lies in its polysaccharide components. During the aging process, these polysaccharides gradually degrade, which contributes to the formation of characteristic flavor profiles in CRP [40]. Umami was the second most prominent taste attribute, with response values ranging from 5 to 6: WZM and HJ had values close to 6, whereas CZG and FJ were closer to 5. Regarding the source of umami, it is primarily derived from free amino acids in CRP. Moreover, as the aging period prolongs, microbial metabolism facilitates the accumulation of free amino acids—enhancing umami intensity and further contributing to the mellow taste profile of CRP [41]. For bitterness, response values ranged from 3 to 5.5, with CZG and WZM exhibiting the highest intensities, while the other cultivars showed slightly lower values (3–4). Consistent with previous reports, the bitter taste intensity of CRP tends to increase during the early stages of storage [41]. Sourness was only detected in FJ, with a response value of 2. Astringency response values were generally low across all CRP cultivars (0–1), with no detectable astringency in DHP, FJ, and HJ. Richness scores were relatively consistent among all samples, averaging around 1.5. Furthermore, response values for astringency and Aftertaste-A were close to zero, while those for sourness, Aftertaste-B, and saltiness were predominantly below zero—indicating that these taste attributes contribute less to the overall flavor profile of CRP.

#### 3.2.2. Statistical Analysis of the Content of Main Flavonoid Components by HPLC

Flavonoids are major components of CRP, contributing significantly to both its pharmacological activities and flavor formation. To evaluate the quality differences in different CRP cultivars, we compared the contents of seven major flavonoids (hesperidin, nobiletin, tangeretin, sinensetin, 4′,5,6,7-tetramethoxyflavone [TMF], 3,5,6,7,8,3′,4′-heptamethoxyflavone [HMF], and 5-demethylnobiletin) in the six cultivars via HPLC analysis, with significant variations observed among different cultivars (Table 1, Figure S6). Hesperidin, the primary flavonoid glycoside in CRP, has been reported to exhibit excellent biological activities [42,43]. Notably, although WZM had a lower total flavonoid content, it showed prominent Hesperidin accumulation (51.1~68.0 mg/g). And hesperidin levels were remarkably high in DHP and FJ (55.3~78.9 mg/g), while relatively lower in ZTH, CZG, and HJ (21.5~4.19 mg/g). The remaining six polymethoxyflavones (PMFs) are common components in Citrus species and have been extensively studied as bioactive ingredients against various diseases [44,45,46,47,48,49]. Overall, ZTH, DHP, and FJ exhibited higher total PMF contents, whereas significantly lower accumulations were observed in other cultivars—particularly WZM. Interestingly, while WZM had extremely low contents of other PMFs with 4–6 methoxy substituents, 3,5,6,7,8,3′,4′-Heptamethoxyflavone (bearing 7 methoxy substituents) exhibited remarkably high accumulation in this cultivar.

#### 3.2.3. Non-Volatile Metabolic Profiles and Flavonoid Preferences Among Different CRP Cultivars

Untargeted LC-MS metabolomic analysis of peels from the six CRP cultivars identified a total of 1289 compounds, with 701 detected in positive ion mode and 588 in negative ion mode. The identified metabolites included 233 flavonoids, 150 heterocyclic compounds, 145 terpenoids, 109 organic acids, 80 coumarins and lignans, 78 ketones, 69 saccharides, 67 lipids, 66 benzene and its derivatives, 61 amino acids and their derivatives, 57 phenolic acids, 43 alcohols and amines, 38 saponins, 33 alkaloids, and 60 other compounds (Table S6). Comparative analysis of the relative proportions of these compound classes across the six CRP cultivars revealed distinct differences in the metabolic profile of WZM. Flavonoids were the dominant compound class in all CRP cultivars: in ZTH, FJ, DHP, CZG, and HJ, flavonoids accounted for over 60% of the total detected compounds. In contrast, flavonoids only constituted 44% of the total compounds in WZM, whereas the contents of coumarins, lignans, terpenoids, amino acids, and lipids in WZM were significantly higher than those in the other cultivars (Figure 5B). To clarify the differentially accumulated metabolites (DAMs) among different cultivars, further multivariate statistical analysis was performed. In the PCA score plot, quality control (QC) samples clustered tightly at the center of the coordinate axes, demonstrating good stability of both the detected samples and the analytical methods. Samples of DHP and HJ overlapped in the upper region of the plot, suggesting similar metabolic profiles between the two cultivars, which is consistent with the results of GC-MS analysis. In contrast, samples of the other four CRP cultivars (CZG, WZM, ZTH, and FJ) were clearly separated into distinct regions, indicating significant differences in non-volatile metabolic profiles among these cultivars (Figure 5C). PLS-DA further confirmed clear discrimination of the six CRP cultivars in the score plot (R^2^Y = 0.396, Q^2^ = 0.0485), validating the existence of significant metabolic differences among the six CRP cultivars (Figure 5D).

Flavonoids were the dominant nonvolatile components. A total of 233 flavonoid compounds were detected in this study, which were classified into 9 subclasses based on structural differences: PMFs, Flavonoid-7-O-glycosides, Flavonoid-3-O-glycosides, Flavonoid C-glycosides, Prenylated flavones, Flavonols, Flavonoid-O-glycosides, Dimethylated flavonoids, and other flavonoids (Figure 5E). As characteristic citrus-derived components, PMFs have attracted extensive attention due to their excellent anticancer activity [50]. Although only 25 PMFs were detected, their total peak area accounted for more than 69% of the total flavonoids—exceeding 85% in CZG, FJ, DHP, HJ, and ZTH. As a representative CRP cultivar with extensive research, CZG has been reported to have high PMF contents in its peel [51]. However, comparative analysis in this study revealed that its flavonoid accumulation is not prominent (Figure S7). Most PMFs are more abundant in DHP and FJ, while ZTH, WZM, and HJ exhibit specific accumulation of PMF compounds that are less abundant in CZG. Considering that the samples used in this study are not long-term stored CRP, and different processing methods have been shown to significantly affect the component contents in citrus peels [52,53,54], the unique processing and aging techniques of CZG may play a crucial role in enhancing its quality. Of course, metabolic differences caused by genetic factors are a key research focus in citrus breeding, so such differences manifested in evolution due to genetic selection are worthy of further exploration in the future.

**Figure 6:**
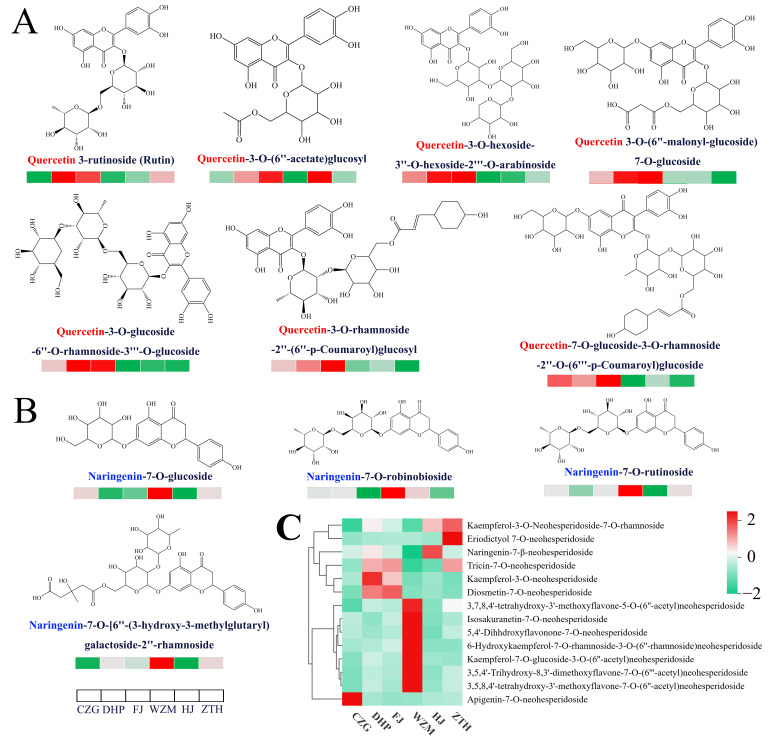
Statistics of characteristic flavonoid glycosides. (**A**) 3-O-flavonoid glycoside type with quercetin as the flavonoid aglycone. (**B**) 7-O- flavonoid glycoside type with naringenin as the flavonoid aglycone. (**C**) Flavonoid glycoside type with neohesperidoside.

The complex evolutionary processes and genetic exchanges among related species in the genus Citrus have led to metabolic differences in flavonoid glycoside substitution patterns across regions and cultivars [55,56]. Citrus peels of different types also exhibit biases in flavonoid glycoside ligands; for instance, flavonoid glycosides in oranges and CZG have been reported to predominantly possess rutinoside ligands [57]. Comparative analysis of metabolic accumulation of flavonoid glycosides with different substitution patterns revealed that most 3-O-flavonoid glycosides (13 compounds) and flavonoid 7-O-glycosides (19 compounds) showed significantly dominant accumulation in DHP and FJ peels, particularly flavonoid 3-O-glycosides with Quercetin as the aglycone (Figure 6A). In WZM, the peak areas of several flavonoid 7-O-glycosides with naringenin as the aglycone were significantly higher than those in other cultivars (Figure 6B). Compared with other cultivars, most glycoside ligands in WZM are of the disaccharide-linked type, with neohesperidoside serving as the predominant ligand for flavonoid glycosides (Figure 6C). Notably, previous studies have indicated that naringin imparts a strong bitter taste, and bitterness formation in citrus peel is closely associated with neohesperidin-related flavonoid glycosides—consistent with electronic tongue results demonstrating that WZM exhibited the strongest bitterness among the six CRP cultivars [58,59]. As one of the important parental lines in citrus breeding, WZM has cultivars widely grown worldwide. Advancing research on the biosynthesis of flavonoids in the peel may provide valuable insights for high-value utilization and functional resource development of citrus [39].

#### 3.2.4. Analysis of Non-Volatile Metabolic Characteristics in Different CRP Cultivars

**Figure 7:**
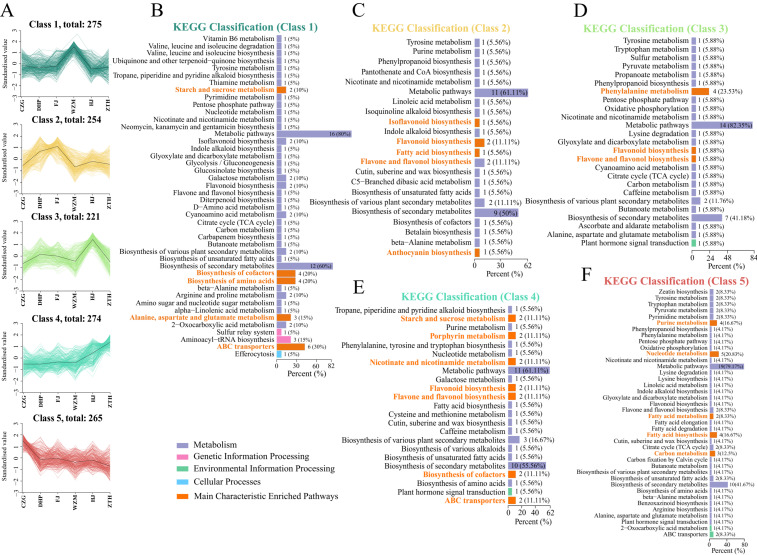
Non-volatile metabolic profiles of six CRP cultivars revealed by K-means clustering analysis and KEGG pathway enrichment. (**A**) K-means clustering classified 1289 non-volatile compounds into 5 classes. (**B**) KEGG pathway enrichment analysis of metabolites in class 1. (**C**) KEGG pathway enrichment analysis of metabolites in class 2. (**D**) KEGG pathway enrichment analysis of metabolites in class 3. (**E**) KEGG pathway enrichment analysis of metabolites in class 4. (**F**) KEGG pathway enrichment analysis of metabolites in class 5.

K-means clustering was performed on the 1289 detected metabolites, revealing five distinct accumulation trends (defined as Class 1–5) that corresponded to the differential metabolic profiles of the six cultivars (Figure 7A). Class 1, one of the five metabolite clusters, consists of 275 compounds, most of which exhibited higher accumulation in WZM. This cluster contains 43 flavonoids and 36 terpenoids, with coumarins and flavonoids as the predominant compounds among those with the largest peak areas. Notably, 3,5,6,7,8,3′,4′-heptamethoxyflavone and hesperidin—reported as major bioactive components in CRP—are part of this cluster. In addition, KEGG pathway enrichment analysis of metabolites in this subclass revealed that the top-ranked pathways included ABC transporters (6 entries), Biosynthesis of amino acids (4 entries), Biosynthesis of cofactors (4 entries), Aminoacyl-tRNA biosynthesis (3 entries), Alanine, aspartate and glutamate metabolism (3 entries), and galactose/starch and sucrose metabolism (2 entries) (Figure 7B, Table S7). Aminoacyl-tRNA biosynthesis and ABC transporters endow WZM with active protein synthesis and substance transport capacities [60,61]. Biosynthesis of amino acids, Alanine/Asp/Glu metabolism, Arg/Pro metabolism, and galactose/starch and sucrose metabolism jointly supply carbon skeletons, nitrogen sources, energy (ATP/NADH), and reducing power (NADPH) [62,63]. Additionally, Flavonoid/Isoflavonoid biosynthesis, Biosynthesis of various plant secondary metabolites, and Biosynthesis of cofactors facilitate the transformation of flavonoids and other functional bioactive components in WZM [64]. This indicates that WZM forms an efficient secondary metabolic synergy among Aminoacyl-tRNA biosynthesis, Amino acid metabolism, Biosynthesis of cofactors, and Flavonoid/Isoflavonoid biosynthesis during the early aging stage. This synergy not only supports the accumulation of functional bioactive components but also provides key precursors for characteristic flavor formation. Meanwhile, amino acids and lipid oxides drive Strecker degradation to promote aroma formation, while efficient flavonoid hydrolysis accelerates the conversion of bitterness to a sweet aftertaste [65].

Class 2 contains 254 compounds that exhibited higher accumulation in DHP and FJ. This cluster has the largest number of flavonoids (70) and phenolic acids (20), while carbohydrates and organic acids are relatively scarce. Most of the compounds with the largest peak areas are PMFs, such as nobiletin, sinensetin, and 3′-demethylnobiletin—bioactive PMFs previously reported to be abundant in CRP [66]. Additionally, although the metabolic profiles of DHP and FJ are generally similar, DHP had lower accumulation levels of certain heterocyclic compounds, coumarins, and amino acids than FJ. This variation may be attributed to differences in soil conditions and climatic factors in their cultivation regions. KEGG pathway enrichment analysis showed that the secondary metabolic networks in DHP and FJ were highly active, with a significant enrichment of bioactive flavonoid components (Figure 7C). The top-ranked pathways included Metabolic pathways (11 entries), Biosynthesis of secondary metabolites (9 entries), Flavonoid/Isoflavonoid/Anthocyanin biosynthesis (5 entries), and Fatty acid biosynthesis (2 entries). Additionally, other pathways—including Nicotinate and nicotinamide metabolism, Pantothenate and CoA biosynthesis, and Biosynthesis of cofactors—are involved in cofactor and energy metabolism. These pathways provide reducing power and activated substrates for flavonoid modification, ester synthesis, and other processes, thereby promoting the transformation of bioactive components in DHP and FJ during the aging stage [62,63,67,68]. Furthermore, Cutin, suberin, and wax biosynthesis, Biosynthesis of unsaturated fatty acids, and Linoleic acid metabolism are responsible for synthesizing cutin, suberin, wax, and unsaturated fatty acids. These substances construct physical barriers, enhance peel mechanical strength and stress resistance, and are conducive to long-term storage [69,70]. Overall, DHP and FJ take PMFs as their core bioactive components, possessing both high accumulation capacity of medicinal bioactive components and peel barrier construction ability, forming a quality characteristic of “prominent efficacy and excellent storability”.

Class 3, consisting of 221 compounds, exhibits high accumulation mainly in HJ. This subclass includes 33 flavonoids, 27 organic acids, and 27 heterocyclic compounds, with terpenoids, flavonoids, and organic acids as the predominant compounds among those with the largest peak areas. Besides Metabolic pathways and Biosynthesis of secondary metabolites, metabolites in this subclass were significantly enriched in Phenylalanine metabolism (Figure 7D)—and phenylalanine serves as the core precursor for secondary metabolites such as phenols, flavonoids, and lignans—indicating that HJ possesses an efficient supply capacity of aromatic amino acids, supporting flavonoid skeleton biosynthesis [71]. Additionally, although most pathways were only enriched with a single metabolite, the characteristic compounds of HJ are widely distributed across multiple functional modules, including flavonoid synthesis, energy metabolism, amino acid allocation, and antioxidant defense. This reflects a high degree of integration in its metabolic network.

Class 4 contains 274 compounds that exhibit significantly high accumulation in ZTH, including 55 flavonoids, 37 heterocyclic compounds, and 33 terpenoids. Metabolites with the largest peak areas are mainly flavonoids and terpenoids; notably, several PMFs (isosinensetin, 5-O-demethylnobiletin, skullcapflavone II) and flavonoid glycosides (swertiajaponin, diosmin, neoeriocitrin) are prominent in this subclass. KEGG pathway enrichment analysis revealed that the top-ranked pathways included Flavonoid biosynthesis, Flavone and flavonol biosynthesis, Porphyrin metabolism, ABC transporters, Starch and sucrose metabolism, Nicotinate and nicotinamide metabolism, and Biosynthesis of cofactors (Figure 7E). This reflects the significant enrichment of flavonoid biosynthesis pathways in ZTH. In conjunction with the synergistic activation of starch/sucrose metabolism, cofactor regeneration, and ABC transport systems, ZTH has constructed a flavonoid metabolic cycle characterized by “efficient synthesis, precise modification, and secure storage”. This specialized yet robust metabolic pattern not only ensures the accumulation of high levels of bioactive components but also endows ZTH with excellent postharvest stability and aging potential.

Class 5, comprising 265 compounds, is characterized by high accumulation predominantly in CZG, including 32 flavonoids, 35 heterocyclic compounds, and 35 terpenoids. Among the metabolites with the largest peak areas, terpenoids, heterocyclic compounds, and amino acids are the main contributors. Further KEGG pathway enrichment analysis of metabolites in this subclass revealed that the top-ranked pathways included Nucleotide metabolism (5 entries), Fatty acid biosynthesis (4 entries), Fatty acid metabolism (2 entries), Purine metabolism (4 entries), and Carbon metabolism (3 entries) (Figure 7F). All these pathways belong to primary metabolism, revealing that CZG exhibits high primary metabolic activity. During the early aging stage, it can synthesize substantial amounts of ATP, NAD(P)H, and membrane lipids, thereby supplying energy and precursors for early aging reactions such as flavonoid modification (e.g., glycosyl hydrolysis) and volatile ester synthesis. This supports the directional transformation of secondary metabolites and accelerates the activation of bioactive components [62,72]. Meanwhile, it also provides ATP/NADPH required for non-enzymatic reactions (e.g., Maillard reaction, transesterification) and residual enzyme activity during aging, resulting in more sufficient flavor evolution and stronger anti-degradation capacity [73]. This accelerates the formation of its quality characteristics, such as aroma enhancement, bitterness reduction, and efficacy improvement.

### 3.3. Evaluation of Antioxidant Activity

**Figure 8:**
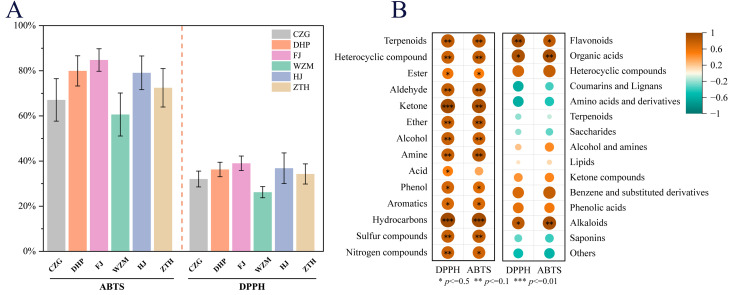
Analysis of antioxidant activity of six CRP cultivars. (**A**) Results of antioxidant activity. (**B**) Antioxidant activity assays of six CRP varieties and their correlations with volatile (**left**) and non-volatile (**right**) metabolites. A vertical red dashed line divides the bar chart into ABTS (**left**) and DPPH (**right**) groups. In panel (**B**), the color gradient represents correlation coefficients ranging from −1 to 1, and the size of each circle is proportional to the absolute value of the correlation coefficient. Significance levels are marked as * *p* ≤ 0.5, ** *p* ≤ 0.1, *** *p* ≤ 0.01.

Antioxidant activity is one of the intrinsic mechanisms underlying most diseases. CRP is rich in flavonoids and phenolic acids, exhibiting considerable antioxidant activity [74]. The DPPH free radical scavenging assay typically reflects the antioxidant capacity of hydrophobic organic compounds, whereas the ABTS assay exhibits sensitivity to both water-soluble and liposoluble antioxidants. Hydrophilic components showed stronger antioxidant activity, indicating that water-based CRP products exhibit high antioxidant potential (Table S8). Three cultivars (DHP, FJ, and HJ) showed higher antioxidant activity (Figure 8A), which is consistent with previous studies reporting that some ancient native Chinese red tangerine cultivars possess stronger antioxidant capacity [75]. ZTH and CZG followed closely, while WZM exhibited significantly weaker antioxidant activity. Correlation analysis revealed that most VOCs were significantly positively correlated with antioxidant activity (Figure 8B). Additionally, flavonoids, organic acids, and alkaloids showed a significant positive correlation with antioxidant activity (*p* < 0.05). Notably, although metabolomic analysis revealed a higher flavonoid content in ZTH, HJ still exhibited stronger antioxidant activity than ZTH. This finding suggests that certain volatile compounds, organic acids, and phenolic acids present in CRP may contribute to its antioxidant capacity [76]. Furthermore, other components also influence antioxidant activity to some extent; for instance, polysaccharides exhibit certain antioxidant activities, and relevant studies have indicated significant differences in polysaccharide content among different cultivars [77]. Aging is a crucial process for CRP, which can significantly alter its bioactivities and functional properties. However, the inherent differences in metabolic components among different cultivars will further induce changes in their chemical profiles during aging, and the dynamic variations in metabolic differences in various cultivars during the aging process warrant further in-depth investigation [78,79].

## 4. Conclusions

This study systematically characterized the aroma profiles, metabolic differences, and antioxidant activities of six CRP cultivars (CZG, DHP, FJ, HJ, ZTH, and WZM). A total of 455 volatile components and 1289 non-volatile components were identified. Metabolic profile analysis revealed significant cultivar-specific accumulation biases of terpenoids and flavonoids among different cultivars, which markedly affected the flavor characteristics and antioxidant activity of each cultivar. DHP (produced in Southwest China) and FJ (produced in Fujian Province) exhibited similar flavor and metabolic characteristics, with the highest contents of volatile compounds. Most monoterpenoids showed characteristic accumulation, and the synergistic effects of certain aldehydes, terpenoids, and esters endowed them with a unique mixed herbal and woody aroma. Meanwhile, these two cultivars were rich in PMFs and several 3-O-flavonoid glycosides, exhibiting a high capacity for accumulating medicinal bioactive components and strong antioxidant activity. HJ and ZTH (both produced in Jiangxi Province) accumulated abundant sesquiterpenoids. HJ possessed a unique sweet-floral aroma derived from characteristic terpenoids and esters; the distinctive orange aroma of ZTH was mainly attributed to 1-undecanol, (E)-2-undecenal, cis-geraniol, neryl acetate, and (E)-β-damascenone. Both cultivars showed favorable performances in flavonoid metabolism and antioxidant activity. Furthermore, our study found that CZG exhibited significant accumulation of certain monoterpenoids, which contributed to the production of herbal aroma; its unique fresh and herbal odor stemmed from the synergistic effects of various VOCs. In particular, methyl anthranilate, α-farnesene, and 3-methyl-1-phenylpentan-3-ol played crucial roles in the formation of its distinctive aroma. In contrast to other cultivars, CZG had no prominent accumulation of flavonoids, while its primary metabolism was highly active. This trait not only facilitated the secondary metabolic transformation of CZG during the early aging stage, accelerating quality formation, but also promoted flavor development. WZM had significantly lower contents of volatile components and flavonoids, especially a remarkable reduction in PMF content. Nevertheless, it exhibited characteristic accumulation of 7-O-flavonoid glycosides with naringenin as the aglycone. Moreover, the high accumulation of most flavonoid glycosides with neohesperidoside glycosyl groups in this cultivar enhanced the formation of its bitter taste.

From the perspective of industrial application, apart from widely commercialized CRP cultivars such as CZG with large market shares, other varieties also exhibit unique metabolic profiles, flavor characteristics, and antioxidant properties that merit further attention from the consumer market. DHP and FJ exhibit high total volatile contents, abundant flavonoids, and strong antioxidant capacity, highlighting their potential for medicinal and health-promoting product development. HJ and ZTH, characterized by rich sesquiterpenoids and distinctive floral orange aromas, can be preferentially developed into natural flavor enhancers and aroma functional food ingredients. WZM, enriched in bitter-tasting flavonoid glycosides, shows promising prospects for specialized bitter-flavored functional foods for targeted health-regulating purposes. Collectively, our results provide solid theoretical support for cultivar evaluation and the tailored development of differentiated CRP-based functional foods, promoting high-value industrial utilization of CRP resources. Future biological validation assays will further verify the functional advantages of elite CRP cultivars, which can offer comprehensive guidance for cultivar utilization, targeted flavor improvement, and the development of high-value CRP functional products.

## Figures and Tables

**Table 1:** Contents of seven major flavonoids in six CRP cultivars (mg/g, *n* = 3).

Sample	Hesperidin	Nobiletin	Tangeretin	Sinensetin	TMF	HMF	5-Demethylnobiletin
CZG	24.8 ± 3.2	2.5 ± 0.1	1.7 ± 0.5	0.4 ± 0.0	0.2 ± 0.0	0.4 ± 0.1	0.3 ± 0.1
DHP	64.1 ± 12.9	5.6 ± 0.5	2.2 ± 0.2	0.9 ± 0.1	0.4 ± 0.1	0.3 ± 0.1	0.6 ± 0.1
FJ	63.9 ± 8.1	6.6 ± 1.6	3.0 ± 0.4	1.1 ± 0.3	0.7 ± 0.1	0.4 ± 0.1	0.6 ± 0.1
HJ	36.7 ± 5.0	5.1 ± 0.4	2.9 ± 0.2	0.3 ± 0.1	0.3 ± 0.0	0.2 ± 0.1	0.8 ± 0.1
WZM	59.0 ± 8.5	0.5 ± 0.1	0.2 ± 0.1	0.2 ± 0.0	0.1 ± 0.0	0.7 ± 0.1	0.2 ± 0.0
ZTH	37.6 ± 5.6	5.7 ± 0.4	2.5 ± 0.0	0.9 ± 0.0	0.7 ± 0.0	0.5 ± 0.1	0.9 ± 0.1

## Data Availability

Data will be made available on request.

## Supplementary Materials

The following supporting information can be downloaded at https://www.mdpi.com/article/10.3390/foods15122090/s1, Figure S1. OPLS-DA Score Plot and Permutation Test Plot for the ZTH versus Other CRP Cultivars. (A) CZG vs. ZTH; (B) DHP vs. ZTH; (C) FJ vs. ZTH; (D) HJ vs. ZTH; (E) WZM vs. ZTH; Figure S2. OPLS-DA Score Plot and Permutation Test Plot for the CZG versus Other CRP Cultivars. (A) DHP vs. CZG; (B) FJ vs. CZG; (C) HJ vs. CZG; (D) WZM vs. CZG; (E) ZTH vs. CZG; Figure S3. OPLS-DA Score Plot and Permutation Test Plot for the DHP&FJ versus Other CRP Cultivars. (A) CZG vs. DHP&FJ; (B) HJ vs. DHP&FJ; (C) WZM vs. DHP&FJ; (D) ZTH vs. DHP&FJ; Figure S4. OPLS-DA Score Plot and Permutation Test Plot for the HJ versus Other CRP Cultivars. (A) CZG vs. HJ; (B) DHP vs. HJ; (C) FJ vs. HJ; (D) WZM vs. HJ; (E) ZTH vs. HJ; Figure S5. OPLS-DA Score Plot and Permutation Test Plot for the WZM versus Other CRP Cultivars. (A) CZG vs. WZM; (B) DHP vs. WZM; (C) FJ vs. WZM; (D) HJ vs. WZM; (E) ZTH vs. WZM; Figure S6. Determination of Seven Major Flavonoids in Six CRP Cultivars by HPLC; Figure S7. Structural formulas and peak area comparisons of 25 PMFs. Red boxes denote higher accumulation in DHP and FJ, blue boxes in ZTH, and green boxes in WZM; Table S1. Sampling location information and cultivar characteristics of six CRP cultivars; Table S2. The GC-E-nose analysis results of six CRPS cultivars; Table S3. Statistics on the relative content of 455 VOCs in 18 CRP samples; Table S4. Characteristic Distribution and Aroma Description Statistics of 130 Terpenoids in Six CRP Cultivars; Table S5. Statistics of rVAO Values of Key VOCs Contributing to Aroma Formation in Different CRP Cultivars; Table S7. Statistics of peak areas of 1289 metabolites in 18 CRP samples; Table S8. KEGG enrichment analysis results of differential metabolites in Six CRP cultivars; Table S9. Evaluation results of antioxidant activity of six CRP cultivars (*n* = 3).

## Abbreviations

The following abbreviations are used in this manuscript:

CRP	Citri Reticulatae Pericarpium
CZG	Citrus *reticulata* ‘Chachi’
DHP	*C. reticulata* ‘Dahongpao’
FJ	*C. reticulata* ‘Tangerina’
WZM	*C. reticulata* ‘Unshiu’
HJ	*C. reticulata* cv. Sanhuhongju
ZTH	*C. reticulata* cv. Zhangshuensis
PCA	Principal Component Analysis
OPLS-DA	Orthogonal Partial Least Squares Discriminant Analysis
VOC	Volatile Organic Compounds
OAV	Odor Activity Value
PMF	Polymethoxyflavone
DEM	Differentially Expressed Metabolites
