# Supplementary material for: Integrated Flavoromics and Metabolomics Reveal Aroma and Bioactive Characteristics of Six Mainstream Citri Reticulatae Pericarpium Cultivars
Source: Foods. 2026 Jun 10;15(12):2090. doi: 10.3390/foods15122090 (PMC13298272; doi:10.3390/foods15122090)
Supplement: Supplementary file 1 [file foods-15-02090-s001.zip › Supplementary Data Figure S1-S7.pdf]

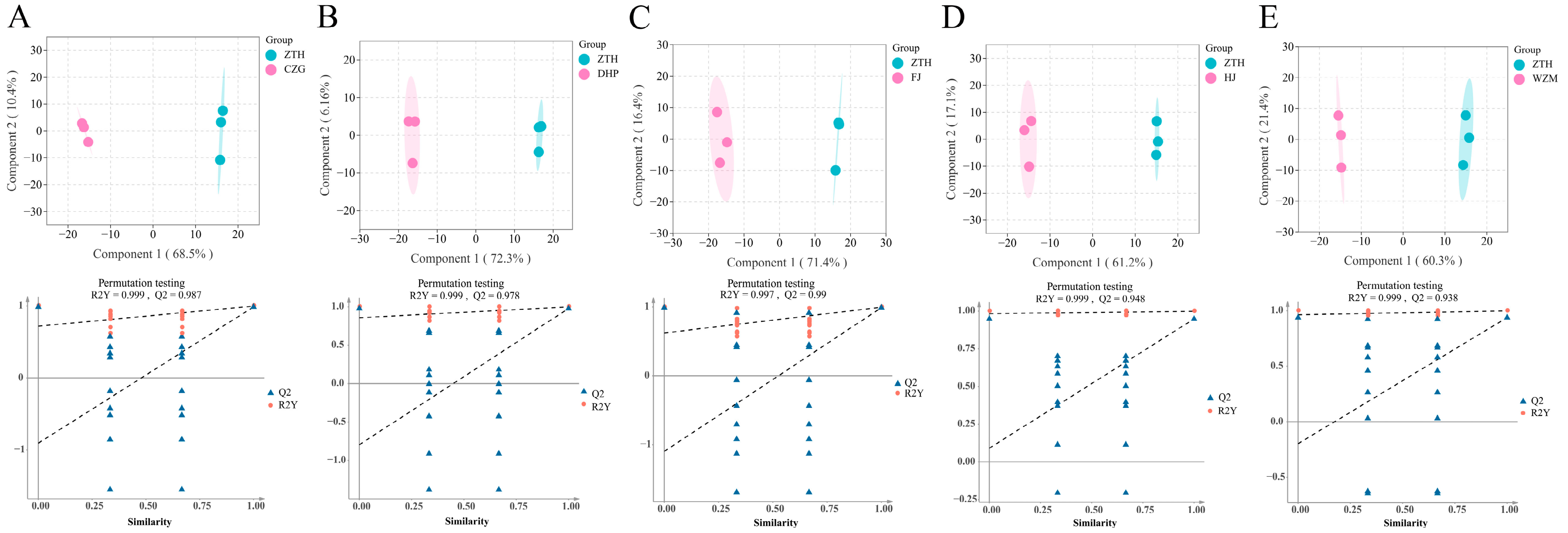

Figure S1 OPLS-DA Score Plot and Permutation Test Plot for the ZTH versus Other CRP Cultivars. (A) CZG vs ZTH; (B) DHP vs ZTH; (C) FJ vs ZTH; (D) HJ vs ZTH; (E) WZM vs ZTH.

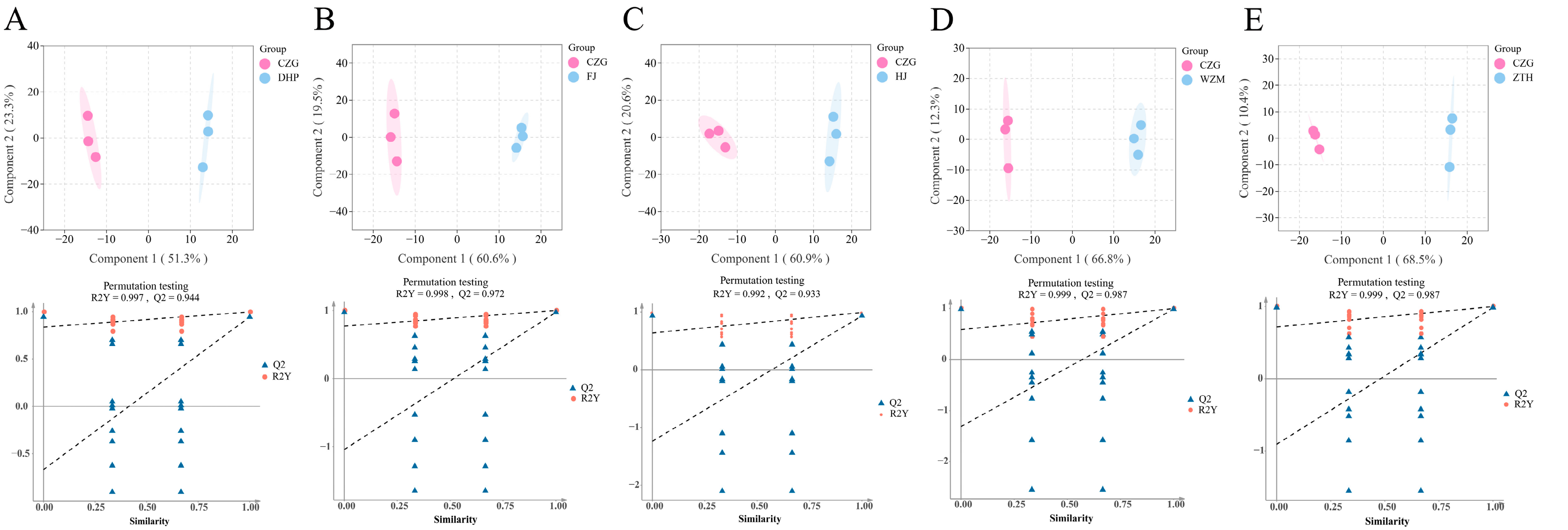

Figure S2 OPLS-DA Score Plot and Permutation Test Plot for the CZG versus Other CRP Cultivars. (A) DHP vs CZG; (B) FJ vs CZG; (C) HJ vs CZG; (D) WZM vs CZG; (E) ZTH vs CZG.

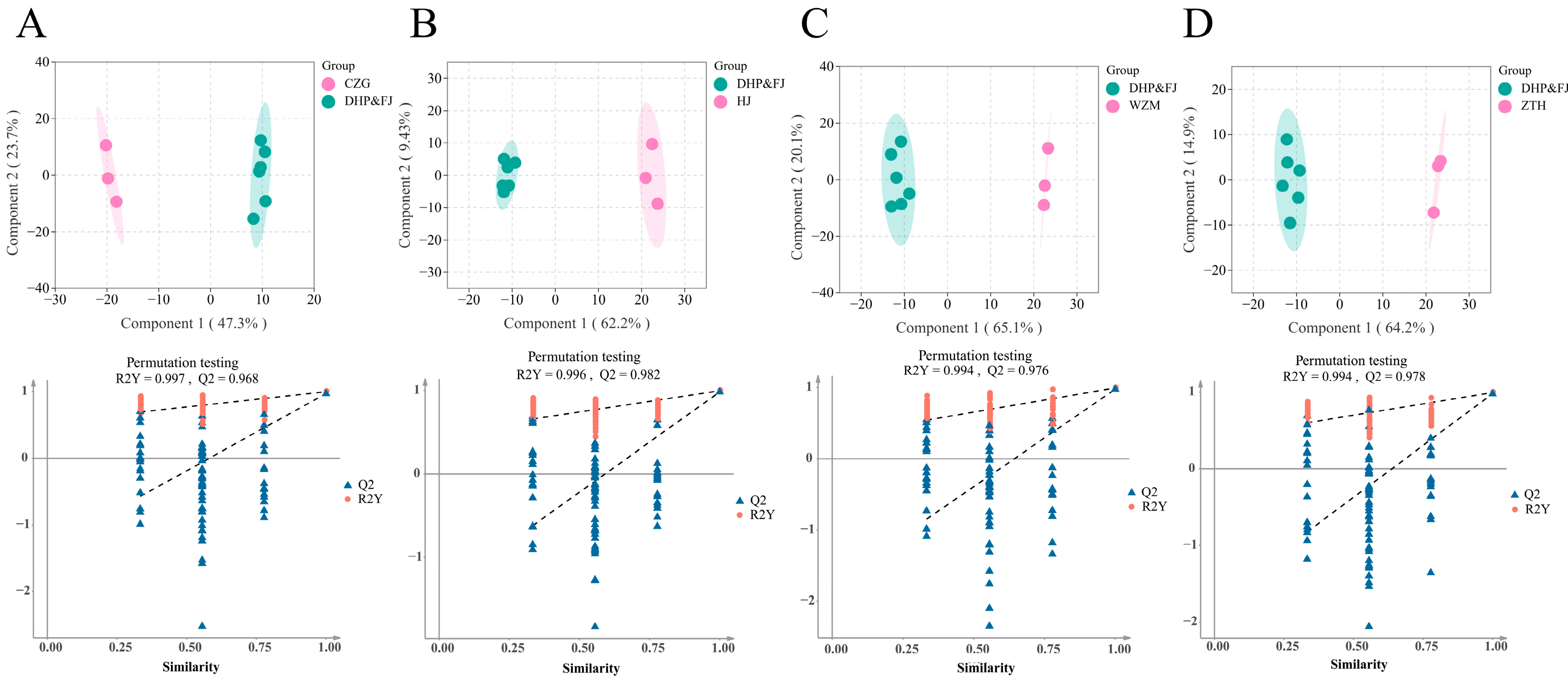

Figure S3 OPLS-DA Score Plot and Permutation Test Plot for the DHP&FJ versus Other CRP Cultivars. (A) CZG vs DHP&FJ; (B) HJ vs DHP&FJ; (C) WZM vs DHP&FJ; (D) ZTH vs DHP&FJ.

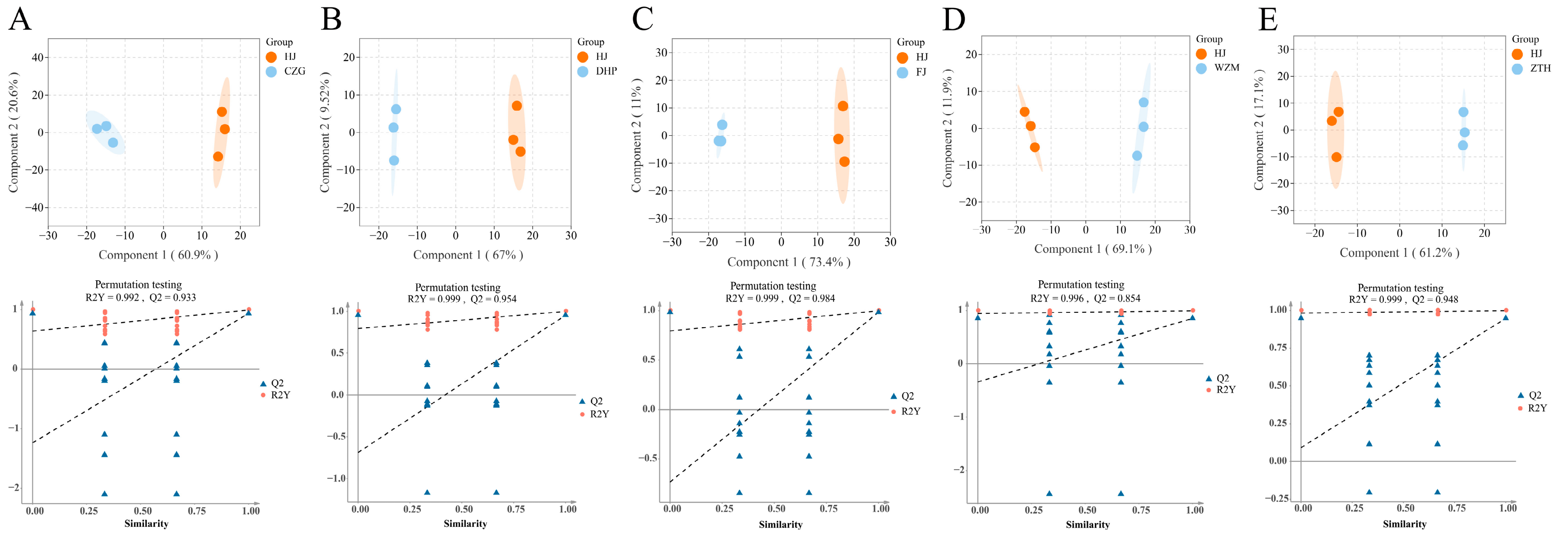

Figure S4 OPLS-DA Score Plot and Permutation Test Plot for the HJ versus Other CRP Cultivars. (A) CZG vs HJ; (B) DHP vs HJ; (C) FJ vs HJ; (D) WZM vs HJ; (E) ZTH vs HJ.

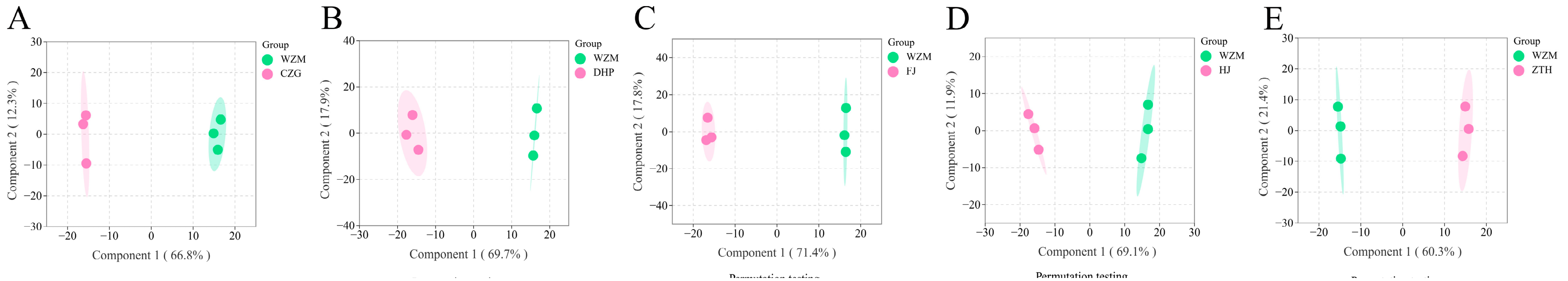

Figure S5 OPLS-DA Score Plot and Permutation Test Plot for the WZM versus Other CRP Cultivars. (A) CZG vs WZM; (B) DHP vs WZM; (C) FJ vs WZM; (D) HJ vs WZM; (E) ZTH vs WZM.
